# Toward workforce integration: enhancements in adaptive behaviors and social communication skills among autistic young adults following vocational training course

**DOI:** 10.3389/fpsyg.2024.1392672

**Published:** 2024-12-11

**Authors:** Yael Lousky, Efrat Selanikyo, Gila Tubul-Lavy, Esther Ben-Itzchak

**Affiliations:** ^1^Bruckner Center for Autism Research, Ariel University, Ariel, Israel; ^2^Ono Academic College, Kiryat Ono, Israel; ^3^Bruckner Center for Autism Research, Department of Communication Disorders, Ariel University, Ariel, Israel

**Keywords:** autism, young adults, employment, vocation, social communication, adaptive behavior

## Abstract

**Background:**

Cognitively able autistic adults demonstrate low rates of employment due to social and vocational challenges. The current study aimed to examine changes in various areas among autistic young adults who participated in the ‘Roim Rachok’ (‘Looking Ahead’ in Hebrew) Training Course (RRTC). The course prepares young autistic adults for integration into military service as vocational soldiers.

**Methods:**

The study included 49 autistic participants who completed the RRTC in one of three vocational fields: Digital (*n* = 19), Technical (*n* = 9), and Visual (*n* = 21). Evaluations at the beginning and end of the course included adaptive behavior (Adaptive Behavior Assessment Scale 2^nd^ Edition [ABAS-II]), autism symptom severity (Social Responsiveness Scale 2nd Edition [SRS-II]), and communication skills (Faux Pas; Empathy Quotient [EQ]; Friendship Quality Scale; Conversation task based on Yale *in vivo* Pragmatic Protocol [YiPP]).

**Results:**

The results revealed a significant Time effect for the self-reported ABAS-II conceptual, social, and practical subdomains, EQ empathy quotient subdomain, Faux Pas, and SRS-II social communication interaction scores. Accordingly, participants reported increasing their adaptive skills, emotional empathy, and the ability to detect and interpret awkward statements, and decreased in their social communication interaction symptoms, following the RRTC. No significant Time x Group interaction was found for any of the examined measures, meaning similar trends were observed in all three vocational groups.

**Conclusion:**

Following the RRTC, participants reported significant improvements in areas essential for their future integration as soldiers in the military and as employees in the vocational world. Implications of the study findings are discussed.

## 1 Introduction

Young adults encounter a new and challenging situations as they pass from adolescence and high-school environment into adulthood (Stroud et al., 2015). For autistic young adults, including those who are cognitively able, the transition into adulthood is far more challenging (Taylor et al., 2022). Autism spectrum disorder (ASD; DSM 5) is a neurodevelopmental disorder characterized by social communication/interaction impairment and restricted/repetitive behaviors, interests, or activities (American Psychiatric Association, 2013). As approximately 67–70% of those with ASD have normal or above normal intelligence (Christensen et al., 2016; Maenner et al., 2021). Turning into adulthood, young autistic adults with normal cognition often find that their diagnostic characteristics adversely impact their ability to assimilate into unsupported neurotypical adult environments such as the vocational world (Baldwin et al., 2014; Chen et al., 2015; Frank et al., 2018; Krzeminska and Hawse, 2020; Lorenc et al., 2018).

Employment significantly influences an individual’s quality of life and identity, involving social integration, contribution to society, decision-making, and reduced dependence on public programs (Scott et al., 2013; Roux et al., 2013). However, the workforce transition for autistic individuals often occurs in environments prioritizing productivity and efficiency over accommodating their unique needs (Pezzimenti, 2004; Bishop-Fitzpatrick et al., 2016). Despite cognitive abilities, only 37–55% of autistic adults obtain employment, often in part-time or underpaid positions (Alverson and Yamamoto, 2017; Chen et al., 2015). Additionally, 22% work below their educational capacity (Frank et al., 2018). Academic education, typically seen as crucial for workforce integration, does not guarantee successful employment for cognitively able autistic young adults (Chen et al., 2015; Taylor et al., 2015). Given these challenges, it becomes imperative to explore ways to enhance employment opportunities for autistic individuals, as employment is closely tied to well-being in adulthood and fulfills crucial social, financial, and psychological needs (Wehman et al., 2014; Scott et al., 2019). Prior research has identified barriers, as well as facilitators, to workforce integration among autistic adults including personal, environmental, and work factors (Harmuth et al., 2018). Among the person-related factors, adaptive behavior and communication skills emerge as primary barriers to employment. Adaptive behavior is comprised of behaviors essential to independent living – daily living skills, self-care, work skills (Harrison and Oakland, 2003). Higher levels of adaptive skills were found associated with optimal outcomes among autistic adults, while poor adaptive behaviors contributed to lower employment even in the presence of normal to high cognitive ability (Farley et al., 2009; Duncan and Bishop, 2015). Lack of social communication skills has also been found to be a primary barrier to employment success for autistic individuals (Black et al., 2020; Chen et al., 2015; Chiang et al., 2013; Harmuth et al., 2018; Holwerda et al., 2012). To achieve and maintain employment, autistic young adults require extensive, customized training in adaptive behaviors and social skills in preparation for the social codes, expectations and ethics essential for adult work life (Müller et al., 2003; Scott et al., 2019; Baker-Ericzén et al., 2022). In addition, employers should make efforts to understand the diverse and sometimes unique qualities of the cognitively able autistic population and attempt to utilize these qualities to meet workplace needs (Bury et al., 2021). In an ideal workplace, employers would recognize both behavioral challenges as well as special skills that autistic individuals may have such as particular cognitive strengths and interests, hyper-attention to details, visual hyper-focus, scientific attitudes, and a developed sense of fairness (Black et al., 2020; Bury et al., 2020). Many researchers and autism advocates have emphasized the potential for success of these individuals particularly in jobs within the technology industry such as programming, information technology, software testing, and cyber-ware (Wei et al., 2013). Adopting strategies that foster and implement appropriate support systems would create a mutually beneficial outcome: autistic employees would gain a supportive and rewarding work environment, while employers would benefit from a skilled, productive, and loyal workforce (Harvery et al., 2021; Hendricks, 2010).

Over the last decade, numerous inquiries have systematically examined the impact of occupational training on essential workplace adaptation skills, particularly those pertaining to social communication, as documented in Appendix A. Two primary models of vocational supported skills interventions have been recognized: (1) Vocational soft skills (social communication, executive functioning) training groups, conducted separately from workplaces (Baker-Ericzén et al., 2018; Gorenstein et al., 2020; Moody et al., 2022; Oswald et al., 2018; Sung et al., 2019; Turner-Brown et al., 2008). (2) Internship programs that integrate vocational soft skills support within workplace settings (Wehman et al., 2020, 2023; Hillier et al., 2008).

Several studies employed pre-post designs (Baker-Ericzén et al., 2018; Sung et al., 2019; Moody et al., 2022), while others utilized randomized control studies (Gorenstein et al., 2020; Turner-Brown et al., 2008; Oswald et al., 2018; Wehman et al., 2023). The study group (undergoing intervention) in vocational soft skills groups had relatively small participant numbers (*N* = 6–22), and the study populations exhibited wide age ranges (e.g., 18–55 years - Turner-Brown et al., 2008; 18–38 years - Oswald et al., 2018; 18–45 years - Gorenstein et al., 2020). Disparities were also noted in program duration (8–24 weeks), with intensity typically set at once a week (Baker-Ericzén et al., 2018; Sung et al., 2019; Gorenstein et al., 2020; Turner-Brown et al., 2008). Improvement in social cognition and empathy skills (Turner-Brown et al., 2008; Sung et al., 2019; Gorenstein et al., 2020), enhanced communication skills (Baker-Ericzén et al., 2018), and improvement in adaptive skills (Oswald et al., 2018) were reported. Supported internship programs (Wehman et al., 2020, 2023) conducted a nine-month randomized controlled trial with an intervention group (*n* = 79) and a control group (*n* = 25), measuring outcomes through post-internship employment rates and income levels. Another important and often overlooked aspect of supportive employment interventions in both models is the professional team that customizes and administers the intervention. Facilitators are mostly recruited from special education, occupational therapy, or psychology fields and have had experience working with the autistic population (Baker-Ericzén et al., 2018; Moody et al., 2022; Sung et al., 2019; Wehman et al., 2020, 2023). Friedman et al. (2019), however, recommended the involvement of speech and language pathologists when the focus of the intervention is social communication skills in the workplace.

Our study aligns with the internship model, examining a vocational training program that utilizes participants’ strengths while providing extensive adaptive skills and social communication support. This program was developed in Israel in collaboration with the Israeli military (Gal et al., 2015). Prior studies in this category typically focused on outcomes such as employment rates and income levels without evaluating adaptive behavior and social skills following the intervention. Our study aims to contribute to the literature by assessing changes in adaptive behavior and social skills following the intervention, as these are the core skills the program seeks to enhance, using standardized measures for evaluation.

### 1.1 Ro’im Rachok (‘looking ahead’) program

In Israel, military service is compulsory, representing a crucial milestone for high-school graduates. While it often serves as the initial step toward future employment for neurotypical individuals, those diagnosed with autism encounter challenges, with only a minority integrated into the IDF (Israeli Defense Forces). Recognizing the increasing prevalence of autism, the IDF collaborated with autism specialists in 2013, resulting in the creation of the Ro’im Rachok Program (RRP). This innovative program envisions a mutually beneficial relationship: cognitively able autistic individuals gain valuable military experience aligned with their strengths, contributing high-quality service professionals to the IDF’s workforce. The RRP focuses on training, supporting, and integrating autistic young adults into technological divisions of the military as well as preparing them for future civilian career options in technological fields. The primary aim of the IDF is to ensure national security while fostering inclusivity and diversity within its ranks. To our knowledge, IDF is the only military in the world to formally integrate autistic young adults in the context of a support program. To date, more than 350 autistic individuals have been recruited via the RRP into the IDF, serving in different 27 military units. The RRP support team is comprised of experts in communication disorders, occupational therapy, and psychotherapy who collaborate to create a distinctive transdisciplinary support model. RRP soldiers (and their commanders) are provided support from the program during active military service, as well as during subsequent civilian employment. The transdisciplinary model focuses on imparting work skills, communication skills, independence skills, and guidance on emotional regulation, in addition to the technical military training. This model is constructed to prepare each participant for the social and behavioral conduct required both in the military and in the workplace. The RRP program involves five stages: (1) screening and allocation, (2) a training course, (3) a trial period within designated IDF units, (4) formal IDF recruitment and military service, and (5) transition to employment in the civilian workforce. Six relevant vocational fields were identified as likely to match the most common characteristics and capabilities of autistic people that are needed by the IDF. These six vocational fields fell into three main tracks (Digital, Technical, and Visual) that shared common skill sets. The Digital track consisted of Software Programming and Data Analysis fields, the necessary skill set for which included English language mastery, detail orientation, high analytical sense, and systematization and technological skills. The Technical track consisted of Electronics and Personal Computer (PC) support fields, the skill set for which included high fine motor skills, technical abilities, problem-solving ability, and a service orientation. The Visual track consisted of Aerial Photo Analysis and Geographic Information Systems (GIS) fields, the skill set for which included visual perception, 3D vision, computer proficiency, high focus ability, and topographical perception.

The current study focuses on the Roim Rachok Training Course (RRTC), the second stage of the RRP. The study addresses several gaps in the literature of vocational preparatory programs tailored for autistic individuals; Firstly, the investigation delves into an intensive preparatory course, distinguishing itself by integrating adaptive behavior and social communication enhancement within a vocational training environment —an approach distinct from the prevalent focus on skills group intervention programs in extant studies. Second, we conducted a comprehensive assessment encompassing various social communication aspects: Theory of Mind, empathy, conversation skills and adaptive skills. Moreover, Most studies evaluated efficacy predominantly relying on self-reported questionnaires while this assessment utilizes a combination of standardized self-report questionnaires and specific tests. Importantly, this research uniquely positions itself to scrutinize alterations and variations in these domains across distinct vocational course fields, thereby capturing the diverse aptitudes prevalent among autistic participants.

### 1.2 Objective of the current study

We aimed to compare possible changes in adaptive behavior and communication skills between three vocational tracks (Digital, Technical, Visual) following RRTC. We hypothesized there would be an increase in adaptive skills and communication abilities, and a decrease in autism characteristics level, following the RRTC. Nevertheless, since the study is innovative in examining this specific program in comparing three vocational tracks among autistic young adults, no direction in the differences between the groups was hypothesized.

## 2 Materials and methods

This research is a pre-post interventional study design (Thiese, 2014), comparing data from two timepoints (pre- and post-RRTC) for three vocational groups.

### 2.1 Participants

The participants for the current study were 56 Jewish Israeli autistic young adults who attended the RRTC between April 2019 and April 2020. All met RRP screening and allocation criteria including: (1) re-confirmation of ASD diagnosis- severity level one (APA, DSM-V, 2013) by a licensed psychiatrist, (2) independence in ADL (activities of daily living), (3) literacy abilities, (4) basic independence in social communication, (4) ability to appropriately handle classified military materials, (5) approval to volunteer by the IDF mental health authorities, and (6) suitability for one of six RRP vocational fields. Two participants were excluded as they did not meet the study’s criterion for verbal ability. Two other participants were withdrawn because they did not complete the full RRTC, and three others requested the exclusion of their data due to personal reasons. Ultimately, the study cohort consisted of 49 cognitively able autistic young adults (47 males, 2 females) with an age range of 18 to 23 years (*M* = 18.74, *SD* = 1.65). The Digital group included 19 participants: Software Programming (9) and Data Analysis (10). The Technical group included 9 participants: Electronics (5) and PC Support (4). The Visual group included 21 participants: Aerial Photo Analysis (11) and GIS (10).

### 2.2 Procedures

The research took place from 1.4.2019 to 30.4.2020 and was approved by the Institutional Review Board of the Ariel University (Protocol Number AU-HEA-EBI-20190206) and the Research Ethics Board of the IDF (Protocol Number- 1980). An additional inclusion criterion was a verbal score within two *SD* on the WAIS-III vocabulary subdomain (Wechsler, 2008) to ensure appropriate verbal competence to undertake the study tasks. At the beginning of the RRTC the participants received an explanation about the study and were asked to provide signed informed consent if they wished to participate. It was clarified that participation in the study was voluntary and that they could discontinue their participation at any stage of the study without consequences. All measurements except for the cognitive evaluation (assessed only at baseline) were taken at two timepoints: the first and last day of the RRTC (T1, T2).

### 2.3 Intervention

#### 2.3.1 Roim Rachok training course

The RRTC prepares autistic young adults for the vocational world through preparation for and integration within the IDF digital, technical, and visual fields. The staff of each group includes a course director and commanders from the designated military units responsible for designing and conveying the course vocational content. Preparation for the military’s vocational daily work routine was executed by a team of speech and language pathologists (SLPs), occupational therapists (OTs), and psychotherapists, all experienced in working with the autistic population. The RRTC was conducted for 12 weeks, consisting of 5 days/week for 7 h/day and it simulated the military’s vocational daily work routine. The course is consisted of two chief elements: vocational training in above specific fields (210 h) and employment preparedness (210 h).

##### 2.3.1.1 Vocational training

Vocational training was taught by IDF trained commanders who were experts in the above fields. The training included theory and practice. The practical training was conducted both individually and as part of a team, similar to a military work setting. The commanders received guidance and support from the RRTC professional team regarding interacting with and teaching the autistic participants. The guidance for the commanders also included methodological adaptions suited for autistic students such as using many organizational graphics, PowerPoint presentations, and a direct manner of speech. The commanders were also guided regarding adapting the study environment to the needs of the autistic participants; for example, they were advised that participants with sensory sensitivities should be provided more individual space around their individual work areas and noise-cancelling headphones.

##### 2.3.1.2 Employment preparedness

The employment preparedness components of the training aimed to help participants acquire the skills required for integration in the IDF as a vocational soldier while simultaneously providing emotional support to cope with the challenges of integrating into a civilian work environment. The topics addressed include social communication, executive functions, personal management, IADL (instrumental activities of daily living) skills, independence skills, working skills, emotional regulation skills and emotional support, yoga and physical workout practice, and discussion about the autistic identity, and the IDF culture and spirit. These preparatory topics were covered in group sessions and are described in detail in Table 1. In cases where the group setting was insufficient, the participant received additional individual guidance.

**Table 1:** Topic and content of employment preparedness.

Topic (# of sessions)	Content details	Conducted by
Social communication (12)	Asking for help; small talk; teamwork; non-verbal communication; Theory of Mind (TOM); texting communication; workplace- appropriate communication; vocational pragmatics; self-advocacy; friendship.	SLP – with commanders
“Get to know your friends” (12 h. preparation, 20 min. Individual presentation)	Preparing a 20-min presentation on a selected area of personal interest; practicing executive functions; time management skills; TOM; pragmatics; giving and receiving feedback.	OT and SLP
Social activities (3 over the training course)	Organizing recreational activities for the course participants; practicing executive functions; communication skills; teamwork.	OT
The autistic identity (12)	Self-identity in light of the diagnosis; semi-structured group discussions regarding the meaning of the diagnosis and its implications for the individual.	OT - with entire staff.
Group support (12)	Sharing emotions; working through emotional issues; processing the course experience.	Psychotherapist
Trust and trustworthiness (12)	Learning about issues of trust; dealing with classified data; integrity; confidentiality.	RR program founder
Current events (12)	Discussing relevant events and issues.	Course director
Yoga (12)	Learning and practicing yoga to promote relaxation and tension release.	Yoga instructor
IDF values and culture (12)	Introducing military jargon; military ethics; typical manners of military communication; IDF ranks and dress code; military time management; communication codes for interaction with commanders/peers.	OT, commander, and SLP
History and Military legacy (12 h. preparation; 20 min. Presentation in pairs)	Preparing a presentation on selected topics from Israel military heritage (guided by a professional team member); practicing executive functions; communication skills.	A retired IDF officer volunteer guided by SLP or OT
Overnight trip (+5 h. pre-trip, 2 h. post-trip)	A two-day trip to sites of Israeli beauty and heritage. Pre-trip preparations were focused on planning and emotional adaptivity. Post-trip was devoted to reflections and feedback.	Entire course staff.

### 2.4 Measurements

#### 2.4.1 Cognitive assessment

##### 2.4.1.1 Wechsler adult intelligence scale (WAIS-III)

The current study utilized WAIS-III verbal and performance scales (Wechsler, 2008). Each scale contains 12 subtests. For the purpose of this study, two subtests (one from each scale) were selected (Lichtenberger and Kaufman, 2009; Silverstein, 1985). The (verbal) vocabulary subtest measures one’s ability to understand, use, and think with spoken language; this subtest represents verbal ability (Raw Scores range 0–60). The (performance) block design subtest measures one’s ability to analyze and synthesize an abstract design; this subtest represents the performance ability (Raw scores range 0–30). Scale scores were transformed into standard scores (*M* = 10, *SD* = 1.5). This study used the Hebrew version of the test by PsychTeck.

#### 2.4.2 Adaptive skills assessment

##### 2.4.2.1 Adaptive behavior assessment scale ABAS 2nd edition- adult version (ABAS-II)

A multidimensional Likert-scale assessment that can be used for self-reports or external reports, the ABAS-II (Harrison and Oakland, 2003) consist of 10 adaptive behavior areas that combine into three main subdomains of adaptive behavior: (1) Conceptual- communication, functional academics, self-direction; (2) Social- leisure and social; (3) Practical- community use, home or school living, self-care, health and safety, and work. Additionally, a General Adaptive Composite (GAC) score is calculated, higher scores indicate better adaptive behavior assessment. Composite scores transform into norm-referenced standard scores (*M* = 100, *SD* = 15). The ABAS-II demonstrates robust reliability across its various psychometric properties, with internal consistency coefficients ranging from 0.85 to 0.98, test–retest reliability coefficients from 0.80 to 0.95, and inter-rater reliability coefficients between 0.80–0.90 (Harrison and Oakland, 2003). This study used the Hebrew version by PsychTeck.

#### 2.4.3 Autism characteristics

##### 2.4.3.1 Social responsiveness scale 2nd edition (SRS-II)- adult version

The SRS-II (Constantino and Gruber, 2012) is a 65-item self and external report Likert-scale assessment of autistic characteristics according to the two criteria of the DSM-V diagnosis: social communication and interaction (SCI) and repetitive restricted behaviors (RRB). SCI is divided into four subdomains: social awareness, social cognition, communication, and social motivation. Higher scores represent more autism related traits. A total score of 59 is considered the clinical cutoff score for autism; 60–65 indicates mild autism traits level, 66–75 indicates moderate autism traits level, and > 76 indicates profound autism. The SRS-2 demonstrates high reliability, with internal consistency (Cronbach’s alpha >0.90) and strong test–retest reliability (*r* = 0.84–0.88) based on correlations between initial and retest scores (Constantino and Gruber, 2012). This study used the Hebrew version of the test (Ben-Sasson and Gal., 2008).

#### 2.4.4 Social-communication skills assessment

##### 2.4.4.1 Faux pas-adult version

The Faux Pas instrument (Baron-Cohen et al., 1999) is a test of interpretation of social situations and social understanding that relies on high order theory of mind (TOM). The test contains 20 short passages describing situations and dialogues from a young adult’s daily life. Ten of the passages contain an awkward or inappropriate statement (faux pas stories) and 10 describe an appropriate statement (control stories). The current study used six faux pas stories (2,4,11,13,14,15) and six control stories (1,3,5,8,10,17), translated to Hebrew with the permission of the authors. The experimenter read the stories aloud while participants followed along with hard copies. The same stories were used at T1 and T2. After reading each passage, the subject is asked eight questions: two comprehension questions (control) and six questions that examine understanding of the social situations including: (1) detection of the faux pas statement, (2) detection of the person who created faux pas situation, (3) understanding of the nature of the inappropriateness of the faux pas, (4) understanding the intentions of the social situation’s players, (5) identifying the false belief, and (6) detection of empathy. Each answer is scored 1 = correct or 0 = incorrect. The sum of each item is calculated separately for the faux pas and control stories. The score range for each item is 0–6, with higher scores indicating better TOM capabilities. The correct identification of faux pas in typically developing individuals ranges between 85 and 90%, while for autistic individuals, it ranges from 49 to 66% (Baron-Cohen et al., 1999; Zalla et al., 2009). Participants’ responses were coded by a licensed SLP blinded to participant diagnosis. A different licensed SLP blinded to participant diagnosis codes 20% of the stories. The kappa statistic between independent ratings was 0.84, indicating very high agreement (Landis and Koch, 1977). When initial disagreements arose, joint discussion was conducted afterward to resolve any remaining discrepancies for consistency in the final dataset.

##### 2.4.4.2 Empathy quotient (EQ)

A self-report Likert-scale questionnaire designed to measure empathy in adults, the EQ (Baron-Cohen and Wheelwright, 2004) consists of 60 statements, with 40 empathy items and 20 control items. A total score ranging from 0 to 80 is calculated, with higher score indicating higher empathy rates. The average score for neurotypical development is 42.1, *SD* = 10.6. In addition, 15 items were loaded onto three factors (Lawrence et al., 2004): (1) Cognitive Empathy Quotient (CEQ)- intellectual apprehension of another’s mental state or perspective, (2) Emotional Empathy Quotient (EEQ)- emotional connection to another person from a shared experience, and (3) Social Skills Quotient (SSQ)- spontaneous use of social skills and intuitive social understanding. Factors scores range is 0–10. Baron-Cohen and Wheelwright (2004) reported a significant negative correlation between EQ and AQ scores (*r* = −0.53) and strong internal consistency (Cronbach’s alpha = 0.92), supporting the EQ’s validity and reliability. This study used Hebrew version of the questionnaire (Milo and Golan, 2006).

##### 2.4.4.3 Friendship qualities scale

A 23-item self-report questionnaire that assesses the quality of one’s relationships with one’s best friends. The Friendship Qualities Scale (Bukowski et al., 1994) measures five components of friendship relations: companionship, conflict, help, security, and closeness. The average scores for neurotypical development are 4.01 (*SD* = 0.38) for Companionship, 2.35 (*SD* = 0.81) for Conflict, 4.19 (*SD* = 0.53) for Help, 4.17 (*SD* = 0.54) for Security, and 4.44 (*SD* = 0.31) for Closeness. The internal reliability of each subscale was examined using two different samples. All subscales for both samples yielded Cronbach’s alpha coefficients between 0.71 and 0.86.(Bukowski et al., 1994).

##### 2.4.4. Conversation task based on Yale *in vivo* pragmatic protocol- YiPP

For the current study, a conversation task based on the YiPP (Simmons et al., 2014) was created. The YiPP protocol measures pragmatic language through a semi-structured conversational task. Within a 30–40-min conversation, the examiner inserts 19 pragmatic measures within four conversational domains (Discourse Management; Communicative Function; Conversational Repair; Presupposition). Each subdomain receive two scores, an Error score – the level of pragmatic performance ranging from 0 to 2 (0 = pragmatic adapted response to 2 = pragmatic non-adapted response) and a Cue score – the level of cueing necessary for the participant to produce the target pragmatic performance ranging from 0 to 6 (6 = a spontaneous and adapted un-cued response and 0 = no response regardless of cue level). Total Error score and total Cue score is also calculated. For the current study, the topics were adjusted for age and interests, and the conversation time was shortened to 5 min. The analysis focused on six pragmatic measures from Discourse Management and Conversational Repair domains: starting conversations, giving background, staying on the topic, giving comments, asking for information and requesting clarification. Score analysis was based on YiPP protocol. Total Error score and total Cue score were calculated. Conversations were recorded in writing and audio to reflect pauses and inflections for coding accuracy. Participant responses were coded by a licensed SLP blinded to diagnosis. Another blinded SLP coded 20% of the stories, and disagreements were discussed among the raters. The kappa statistic between raters was 0.82 (very high; Landis and Koch, 1977) and 0.80 (substantial; Landis and Koch, 1977) for the Error scores and Cue scores, respectively.

### 2.5 Statistical analysis

We conducted the analysis using SPSS version 25 (IBM Corp., 2017). At baseline, the three vocational groups were compared for age, maternal education, IQ scores (verbal vocabulary and performance block design), and autism symptom severity (SRS-II total score) using one-way analyses of variance (ANOVAs). In addition, 2*3 contingency tables were conducted to examine group difference for the categorial demographic data (sex, matriculation exam completion, and presence of comorbidities).

Next, we examined the distribution of the study’s dependent variables at T1 using Shapiro–Wilk test of normality (*p* > 0.05). The ABAS-II, SRS-II, EQ, and Friendship qualities scale subdomain scores were normally distributed while the Faux Pas and conversation task scores were not normally distributed. For the variables that were normally distributed, the homogeneity of the covariance matrix was tested using M Box test and confirmed (*p* > 0.05).

We performed four 3 × 2 multivariate analyses of variance (MANOVAs) (3 Groups × 2 Times) with repeated measures for Time for ABAS-II, EQ, and Friendship Qualities Scale subdomain scores. When Time × Group interactions were significant, simple main effect tests were used. For all significant effects ANOVAs were used. As two SRS-II subdomains (SCI and RRB) were highly correlated >0.7 using Pearson correlation testing, we conducted two separate 3 × 2 ANOVAs with repeated measures for Time for each subdomain separately. For the Faux Pas and conversation task scores, we performed non-parametric Wilcoxon signed-rank testing to examine the change from T1 to T2, as well as Cohen’s Kappa statistic for inter-rater reliability. For analyses where changes were observed, we explored the data to gain a deeper understanding of the distribution of effects. We also generated “change” variables (T2-T1) for the full sample and conducted Pearson correlations to assess relationships among these variables. Additionally, we performed linear regression analyses to predict changes in each variable, using baseline measures (T1) and the SRS total score at T1 as independent variables.

## 3 Results

Table 2 presents characteristics of the three vocational groups including age, years of maternal education, the Wechsler Adult Intelligence Scale (WAIS-III) verbal vocabulary score, the WAIS-III performance block design score (Wechsler, 2008), and the Social Responsiveness Scale (SRS-II) total score (Constantino and Gruber, 2012). Table 3 presents additional characteristics include the percentage of participants who were females, had completed final exams in high school, and had secondary conditions. No significant difference was noted between the vocational groups in any of the parameters.

**Table 2:** Demographics and characteristics among vocational groups.

	Digital *n* = 19	Technical *n* = 9	Visual *n* = 21	*F*	*p*
Age *M* (*SD*)	18.63 (0.91)	19.03 (0.86)	18.71 (2.35)	0.17	0.84
Years of maternal education *M* (*SD*)	0.37 (2.22)	15.89 (3.30)	14.52 (2.44)	1.21	0.31
WAIS-III verbal vocabulary score *M* (*SD*)	12.95 (3.40)	12.77 (1.79)	11.30 (2.61)	1.92	0.16
WAIS-III performance block design score *M* (*SD*)	12.63 (3.11)	10.52 (4.73)	11.17 (3.72)	1.23	0.30
SRS-II total score *M* (*SD*)	70.63 (29.44)	58.41 (23.84)	59.24 (23.30)	1.17	0.32

**Table 3:** Additional demographics, characteristics, and secondary conditions among vocational groups.

	Digital *n* = 19	Technical *n* = 9	Visual *n* = 21	*X* ^2^	*p*
Female (%)	10.5%	0	0	3.29	0.19
Complete high school finals (%)	94.7%	88.8%	100%	4.74	0.36
Secondary conditions (%)					
No secondary conditions	21%	21%	9.5%	4.73	0.09
ADHD	57.9%	33.3%	71.4%	3.81	0.15
Anxiety disorder	10.5%	33.3%	42.8%	5.23	0.10
Depression	15.8%	22.2%	14.2%	0.30	0.86
Learning disability	15.8%	22.2%	23.8%	2.06	0.81
OCD	0	22.2%	38.5%	6.24	0.24

Secondary conditions previously diagnosed according to participants’ personal files.

### 3.1 Adaptive skills

The analysis yielded a significant Time effect [*F*(3,44) = 5.11, *p* = 0.004, *η^2^* = 0.26] but neither a Group effect [*F*(6, 90) = 0.57, *p* = 0.75, *η^2^* = 0.04] nor a Time × Group interaction [*F*(6, 90) = 1.28, *p* = 0.27, *η^2^* = 0.08] were found.

Examining separately each ABAS-II subdomain using 3 × 2 ANOVAs revealed a significant Time effect for all three subdomains (conceptual, social, practical). See Table 4. For all three subdomains, a significant increase in adaptive behavior was noted from T1 to T2. These findings point to improvement in conceptual, social, and practical subdomains of adaptive behavior, as reported by the study participants, independent of their vocational group.

**Table 4:** Means and standard deviations for ABAS-II, SRS-II, EQ and friendship quality scale scores at T1 and T2.

		T1	T2	*F*	*p*	*η*^2^
ABAS-II *M* (*SD*)	Conceptual	95.59 (13.94)	102.35 (14.17)	13.00	0.001	0.22
	Social	89.87 (11.71)	94.89 (14.14)	7.84	0.007	0.15
	Practical	87.49 (15.29)	95.79 (17.23)	14.51	<0.001	0.24
SRS-II *M* (*SD*)	SCI	51.74 (21.29)	47.76 (20.54)	4.39	0.04	0.09
	RRB	11.81 (6.07)	10.88 (6.39)	0.81	0.37	0.02
EQ *M* (*SD*)	EEQ	3.88 (2.29)	4.73 (2.95)	10.08	<0.001	0.18
	CEQ	4.81 (2.38)	5.29 (2.34)	0.21	0.65	0.01
	SSQ	5.24 (2.24)	5.78 (2.50)	2.72	0.11	0.06
Friendship quality scale *M (SD)*	Companionship	3.79 (0.80)	3.69 (0.75)	0.95	0.33	0.02
	Conflict	2.52 (0.73)	2.58 (0.99)	0.63	0.43	0.01
	Help	4.14 (0.57)	4.07 (0.68)	0.05	0.87	0.00
	Security	3.67 (0.54)	3.62 (0.64)	1.62	0.21	0.03
	Closeness	4.17 (0.68)	4.23 (0.83)	0.42	0.52	0.01

### 3.2 Social communication skills

#### 3.2.1 Autism characteristics

The analyses revealed a significant Time effect only for the SRS-II SCI, but not for the SRS-II RIRB, subdomain (see Table 4). No significant Group effect or Time × Group interaction for SRS-II SCI scores [*F*(2,46) = 0.68, *p* = 0.51, *η^2^* = 0.03; *F*(2,46) = 1.55, *p* = 0.22, *η^2^* = 0.06, respectively] or for SRS-II RIRB scores [*F*(2,46) = 0.16, *p* = 0.85, *η^2^* = 0.01; *F*(2,46) = 2.08, *p* = 0.14, *η^2^* = 0.08, respectively] were found. Participants’ self-reported scores reflected a decrease in social communication impairment from T1 to T2 as measured by the SRS-II, independent of their vocational group.

#### 3.2.2 Social cognition

Significant improvements were observed across all six categories of the faux pas stories: Detection, Person, Explanation of Inappropriateness, Intentions, Belief, and Empathy, each with medium effect size. In contrast, no significant changes were found in the control stories (see Table 5).

**Table 5:** Mean scores for the Faux Pas Test and Conversation Task.

		T1	T2	*Z*	p	r
Faux Pas test Faux Pas stories *M* (SD)	Detection	5.34 (0.88)	5.67 (0.67)	−2.84	0.001	0.40
	Person	5.04 (1.03)	5.41 (0.87)	−2.59	0.005	0.37
	Inappropriateness	4.63 (1.42)	5.17 (1.13)	−2.34	0.01	0.33
	Intentions	4.04 (1.44)	4.71 (1.04)	−2.89	0.002	0.41
	False belief	4.79 (1.21)	5.15 (0.95)	−2.03	0.02	0.28
	Empathy	4.81 (1.02)	5.89 (0.82)	−2.07	0.01	0.30
Faux Pas test Control stories *M* (SD)	Detection	4.53 (1.32)	4.61 (1.65)	−0.50	0.32	0.07
	Person	4.53 (1.32)	4.61 (1.65)	−0.50	0.31	0.07
	Inappropriateness	4.31 (1.58)	4.61 (1.65)	−1.02	0.16	0.14
	Intentions	4.55 (1.33)	4.61 (1.45)	−0.45	0.33	0.06
	False belief	5.83 (0.36)	5.78 (0.53)	−0.67	0.27	0.09
	Empathy	5.25 (0.94)	5.22 (1.21)	−0.02	0.49	0.01
Conversation task *M* (SD)	Error score	0.28 (0.26)	0.30 (0.29)	−0.15	0.88	0.02
	Cue score	4.6 (1.17)	4.7 (4.68)	−0.32	0.75	0.04

#### 3.2.3 Empathy

Examination of change over time on EQ scores from T1 to T2 yielded a significant Time effect [*F*(3,44) = 3.39, *p* = 0.03, *η^2^* = 0.19]. No significant Group effect [*F*(6, 90) = 1.66, *p* = 0.14, *η^2^* = 0.10] or Time × Group interaction [*F*(6, 90) = 1.92, *p* = 0.09, *η^2^* = 0.11] was found. We further examined each of the EQ subdomains separately using a series of 3 × 2 ANOVAs. The analyses revealed a significant Time effect only for the EEQ, indicating improvement in emotional empathy from T1 to T2 for all groups, but not for the CEQ or SSQ factors (see Table 4).

#### 3.2.4 Friendship

Evaluation of changes over time on Friendship Qualities Scale scores showed no significant Time [*F*(5,42) = 0.11, *p* = 0.40, *η^2^* = 0.11] or Group [*F*(10,86) = 0.16, *p* = 0.67, *η^2^* = 0.08] effect or a Group X Time interaction [*F*(10,86) = 0.26, *p* = 0.25, *η^2^* = 0.01]. This indicates no significant change between T1 and T2 scores. See Table 4.

#### 3.2.5 Conversation skills

Wilcoxon signed-rank testing did not yield a Time effect for the Total Error score [*Z* = −0.15, *p* = 0.44, *r* = 0.02] or total Cue score [*Z* = −0.32, *p* = 0.37, *r* = 0.04], indicating no change over time between T1 and T2. See Table 5.

Of note, since the Technical group contained the smallest number of participants compared to the other two groups, we re-conducted all the above analyses, comparing only for the Digital and Visual vocational groups. The results were consistent with the previous analyses, showing no difference in the change of the dependent variables between the two vocational groups following the course. Therefore, we decided not to withdraw the Technical group from the analysis and presented the comparison between the three vocational groups.

### 3.3 Change statistics

**Figure 1:**
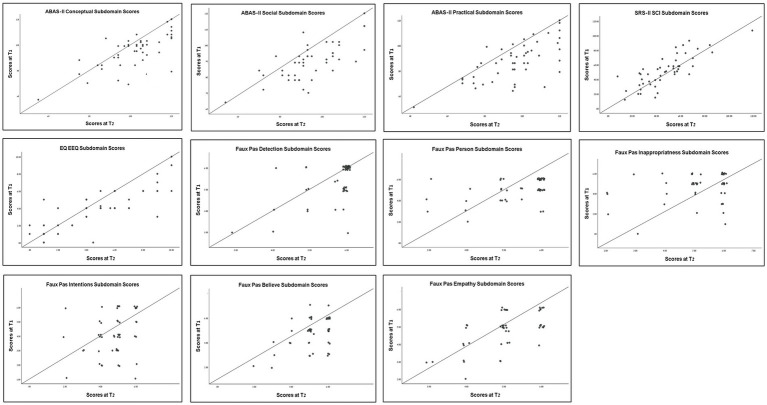
T1 vs. T2 scores across ABAS-II, SRS-II, EQ, and Faux Pas subdomains.

Results revealed an increase in the ABAS-III conceptual, social, and practical subdomain scores, EQ-EEQ, and Faux Pas six subdomain scores as well as a decrease in SRS-SCI score, between T1 and T2 (Overall 11 subdomains). Since no significant differences were observed between the groups, they were combined for the following analyses and were evaluated as a single group. Improvement rates varied across variables. Over half of the participants showed improvements in the SRS-SCI (55.1%), ABAS-II Conceptual (69.4%), Social (61.2%), and Practical (67.3%) subdomains, as well as in the EQ-EEQ subdomain (61.2%) and the Faux Pas Intentions subdomain (53.1%). For the remaining Faux Pas subdomains, 46.9% of participants improved in Inappropriateness, 42.8% in Person, and 38.8% in both Believe and Empathy, while 36.6% showed improvement in Detection.(See Figure 1).

Examining the correlations between the 11 “change” variables required a Bonferroni correction to control for multiple comparisons, adjusting the critical *p*-value to 0.00045. No significant correlations were found among the changes in these measurements (*r* = −0.24 to 0.36, *p* = 0.005–0.97).

In order to understand the individual variability in response to the course and the factors that influenced better progress, we proceeded to perform 11 linear regressions for the measures where improvement was observed. The “change” values (∆ SRS II-SCI, ∆ ABAS Conceptual, Social and Practical subdomains, ∆ Faux Pas subdomains scores, ∆EQ-EEQ scores) served as a dependent variable in each regression, and the baseline value and the SRS-II total scores were used as independent variables. As presented in Table 6 the SRS-II total score did not predict changes in any of the variables, indicating that the initial level of autism had no impact on participants’ improvements. Baseline scores in the ABAS-II Conceptual and Practical subdomains, SRS-SCI, and Faux Pas subdomains significantly predicted changes in these measures. The predictive strength of the baseline measures varied between weak to moderate, with the explained variance (R^2^) ranging from 9% to 59%. Notably, all associations were in a negative direction, indicating that higher initial levels were linked to less improvement across the measures. For the SRS-SCI the baseline scores explained 13% of the variance in the change over time (*R*^2^ = 0.13, *p* = 0.01), with a negative correlation between initial SRS-SCI scores and improvement (*β* = −0.36). In the ABAS-II Conceptual domain, baseline conceptual adaptive behavior explained 14% of the variance (*R*^2^ = 0.14, *p* = 0.01), with a negative correlation (*β* = −0.41) indicating that higher initial scores were associated with less improvement. Faux Pas In the ABAS-II Practical domain, the explained variance was 9% (*R*^2^ = 0.09, *p* = 0.04), and the correlation was also negative (*β* = −0.35). Additionally, all Faux Pas subdomains demonstrated strong, significant negative associations with baseline scores. For the Detection subdomain, baseline detection scores explained 46% of the variance (*R*^2^ = 0.46, *p* < 0.001), with a strong negative correlation (*β* = −0.68). Similarly, baseline scores in the Person subdomain significantly predicted changes, explaining 41% of the variance (*R*^2^ = 0.41, *p* < 0.001) with a strong negative correlation as well (*β* = −0.60). In the Inappropriateness subdomain, the baseline explained 53% of the variance (*R*^2^ = 0.53, *p* < 0.001), and the correlation was notably strong and negative (*β* = −0.77). Baseline scores also significantly predicted changes in the Intentions subdomain, showing a very strong negative correlation (*β* = −0.82) and explaining 59% of the variance (*R*^2^ = 0.59, *p* < 0.001). Baseline scores in the False Belief subdomain significantly predicted changes, with a strong negative correlation (*β* = −0.64) and explaining 48% of the variance (*R*^2^ = 0.48, *p* < 0.001). Lastly, baseline scores in the Empathy subdomain also significantly predicted changes, explaining 44% of the variance (*R*^2^ = 0.44, *p* < 0.001) with a negative correlation (*β* = −0.50). Baseline scores in ABAS-II social subdomain and EQ-EEQ did not significantly correlated with the change in those variables (see Table 6).

**Table 6:** Regression analyses of predictive variables on changes in adaptive behavior and social communication measures (T2-T1).

Change in dependent variable (T2-T1)	Predictive variables	*β*	*R* ^2^	*P*
SRS_SCI	T1 SRS-SCI	−0.36	0.13	0.01
ABAS-II conceptual	T1 ABAS-II conceptual	−0.41	0.14	0.01
	T1 SRS total	−0.11		0.46
ABAS-II social	T1 ABAS-II social	−0.28	0.07	0.09
	T1 SRS total	−0.10		0.13
ABAS-II practical	T1 ABAS-II practical	−0.35	0.09	0.04
	T1 SRS total	−0.14		0.41
EQ-EEQ	T1 EQ-EEQ	−0.21	0.06	0.31
	T1 SRS total	0.01		0.09
Faux Pas Detection	T1 detection	−0.68	0.46	<0.001
	T1 SRS total	0.04		0.70
Faux Pas Person	T1 person	−0.60	0.41	<0.001
	T1 SRS total	0.01		0.40
Faux Pas Inappropriateness	T1 Inappropriateness	−0.77	0.53	<0.001
	T1 SRS total	0.01		0.13
Faux Pas intentions	T1 intentions	−0.82	0.59	<0.001
	T1 SRS total	0.01		0.45
Faux Pas false belief	T1 false belief	−0.64	0.48	<0.001
	T1 SRS total	0.01		0.27
Faux Pas Empathy	T1 empathy	−0.50	0.44	<0.001
	T1 SRS total	0.01		0.09

## 4 Discussion

Given the low employment rates among autistic young adults (Chen et al., 2015; Frank et al., 2018), there is a growing need for tailored support programs to prepare them for the workplace (Wehman et al., 2020). RRTC is an innovative model for training young autistic adults to better integrate into the workforce by first training them to succeed in a military service context. The findings of the current study were positive, indicating that following the RRTC intervention, participants in the different vocational tracks (Digital, Technical, Visual) reported improvements in their adaptive skills, emotional empathy, and showed greater ability to recognize and interpret awkward statements, alongside a reduction in social communication interaction symptoms. Effect size ranges between medium to large across measurements indicating the strength of the observed changes. These skills are crucial for their future integration both as soldiers in the military and as employees in the competitive workforce. Identifying the factors that influenced improvement revealed that neither the vocational group nor the baseline autism level affected changes in the variables.

Notably, adaptive behavior, as measured by ABAS-II, showed significant improvement in all three subdomains: conceptual, social, and practical, each exhibiting a large effect size and a high percentage of improved participants. Higher baseline conceptual and practical adaptive skills correlated negatively with the change in these measures at the end of the course. This can be explained by the relatively high scores at the beginning of the course in the study population that enable only small improvements based on the basic challenges in the autistic study population. However, further improvements were still possible in these domains according to ABAS-II scoring. These positive results in ABAS-II, with strong effects, brought the participants’ scores closer to the normal range (within one *SD*), indicating they were actually gaining these skills during their transition to adulthood. Other studies of vocational support programs have similarly reported enhanced adaptive behaviors among cognitively able autistic adults following interventions, though with a medium effect size (Bonete et al., 2015; Oswald et al., 2018; Connor et al., 2022). Finally it should be noted that the improved self-reported adaptive behavior may reflect the participants’ improved sense of self-efficacy after participating in the RRTC: possibly after completing the course they felt better prepared to tackle the next stage of workforce integration.

Following the RRTC intervention program, significant improvements was also seen in several areas of social communication. Participants reported improvement in their social communication as measured by SRS-II SCI subdomain after the course. Approximately half of the participants reported a decrease in autistic social communication characteristics following the course. Individuals who started with fewer social communication difficulties were able to make more significant gains in this area. Finding ways to improve in these critical areas is a primary goal of intervention programs designed to help young autistic adults transition successfully into adult employment settings. Previous interventions that used self-reported SRS-II to assess effectiveness (Baker-Ericzén et al., 2018; Gorenstein et al., 2020; Sung et al., 2019) reported reductions in SCI with effect sizes ranging from small (Baker-Ericzén et al., 2018; *d* = 0.28) to medium (Sung et al., 2019; *d* = 0.65). Gorenstein et al. (2020) reported no change in self-reported SRS-SCI following their intervention. Notably, our study observed a decrease in SRS-II SCI subdomain scores following the training course, with a medium effect size (η^2^ = 0.09). The higher effect size in our study may be attributed to the internship model, which combined strength-based vocational training with skills enhancement. This approach likely made participants feel more communicative by providing practical experience and support in real work settings, unlike previous studies that focused primarily on skills enhancement alone.

In addition, Faux Pas scores also reflected significant improvements across all six domains tested, with medium effect size, representing the participants’ improved ability to identify and interpret statements inappropriate for adult communication. Improvement was observed in approximately 37–53% of participants, varying by subdomain. Autistic young adults, for whom a chief characteristic of their diagnosis is social communication impairment, often struggle with this. Studies have found that self-reports by cognitively able autistic individuals and reports by their employers indicate that communication and social skills are the primary barrier to successful integration in the workforce (Lorenz et al., 2016; McKnight-Lizotte, 2018). Thus, learning to navigate adult social situations more pragmatically is essential for autistic young adults (Friedman et al., 2019). The changes in Faux Pas subdomain scores were negatively predicted by baseline scores, indicating that participants with higher initial scores exhibited less improvement at T2. This could suggest a ceiling effect, as scores at T2 approached those typically observed in neurotypical development, as reported by Zalla et al. (2009).

Finally, more than half of the participants demonstrated an improvement in emotional empathy. This enhancement, shown by a significant effect size in the Empathy Quotient’s Emotional Empathy subdomain (EEQ), could indicate a deeper emotional connection among participants, likely fostered by the shared, intensive experience of the RRTC program. Possibly, creating tracks for the participants according to their areas of interest and capabilities (Digital, Technical, Visual) created camaraderie based on shared interests rather than sharing only commonality of autistic traits. This notion is strengthened by the fact that each of the vocational track groups improved in this specific parameter. Previous studies have shown that in self-reports, as well as in parental reports of autistic young adults, lack of recognition and utilization of their true interests and aptitudes presented a significant difficulty to workforce integration. Indeed, feeling mismatched, disinterested, and insignificant are likely hindrances to reaching maximum capacity in social skills and adaptive behavior (Bross et al., 2021; Sosnowy et al., 2018). That autistic young adults in previous studies cited science and information technology as characteristic interests accords with the findings of this study in suggesting that learning social skills increase when participants are employed in meaningful settings that are suited to their true interests and strengths.

In Summary, the participants in the RRTC displayed significant improvement in adaptive behavior and social communication aspects over a three-month period in the areas that RRTC focused on. The observed improvements can likely be attributed to several factors, including the intensive support provided by the professional team, the internship model that combines training with ongoing support, the participants’ motivation to succeed in the course and prepare for future military service, and the strength-based approach of the program. It is most likely a combination of some or all of these elements. Additionally, the similar gains observed across the three vocational tracks and the lack of connection to the level of autism suggest that the RRTC had a broad positive impact, regardless of individual aptitudes. However, some areas of improvement were influenced by the participants’ initial functioning levels.

### 4.1 Strengths and applications

Studies examining the results of employment training for young autistic adults have shown varied models of interventions and assessments (Appendix A). However, many of these studies are based on a small sample of participants, which impacts their findings’ generalizability and applicability (Baker-Ericzén et al., 2018; Sung et al., 2019; Gorenstein et al., 2020; Moody et al., 2022; Dean et al., 2022). The population sample in this study is considerable in comparison to the majority of vocational interventions, with 49 participants. Moreover, to the best of our knowledge, this is the first study of an employment intervention that was tailored, conducted, and assessed in a military service setting. The unique setting combined with specialized knowledge and skill content imparted during the training makes this study highly exceptional. Most militaries around the world do little or nothing to support the integration of autistic citizens; in stark contrast, the IDF was open to establishing a program that utilizes the unique strengths of autistic individuals. Thus, the RRTC is a strength-based program, the aim of which is to integrate these young adults into highly respected and coveted units, while other programs geared toward placements in commonplace or task-based employment settings (Wehman et al., 2020;^2023^). An additional aspect unique to this employment training model is the vocational training the participants received in specialized technological areas that are highly transferable to higher-paying positions in the competitive marketplace. This is a particularly meaningful accomplishment for this population which statistically suffers from financial instability and poor earning power. Coupled with the improved social and communication skills and adaptive behaviors observed post-RRTC, the participants appear to have received a comprehensive, intensive preparation for the real-life employment scene.

Another major strength of the RRTC is its integrative team of professional facilitators. The team included speech and language pathologists, occupational therapists, and psychotherapists, who were highly experienced and well-trained to deeply address a broad array of relevant topics and offer a strong support infrastructure to the participants and their IDF commanders. Moreover, combining the contributions of the facilitation team with vocational training and military acculturation provided by the IDF commanders created a very wide base of support and knowledge for the participants.

### 4.2 Limitations

There were several study limitations worth noting. According to IDF policy during the years this study was conducted, autistic young adults who wish to serve in core vocational fields, can only be integrated via participation in Roim Rachok Program, which includes RRTC. Due to this policy and the fact that there was no waiting list for the RRP, we were unable to establish a control group that did not undergo the intervention. The absence of a control group hinders a more conclusive determination that RRTC participation led to the positive outcome. Nevertheless, the limited three-month duration of the course makes it improbable to attribute the results solely to maturation. It is more plausible that the impactful experience stemmed from participating in a course with intensive professional support.

Another limitation is the lack of external assessments to compare with the participants’ self-reported assessments; prior to the start of the course (T1) the facilitation team and IDF commanders were not yet familiar with the participants and therefore could not properly evaluate them. However, the improvement in understanding of Faux Pas stories, which was not self-reported but was rated by experts, may validate the improvement shown in self-reported measures of social communication. A longer study timeline in the future would provide more opportunities to assess the effects of the program and allow for both external assessments and self-reports.

### 4.3 Future studies

This study is the first to examine the efficacy of Roim Rachok Program by evaluating the changes in adaptive behavior and social communication of autistic soldiers over full military service. Going forward, comparisons between the autistic soldier groups and groups of control soldiers with typical development will be essential, as will the collection of external reports from IDF unit commanders regarding the autistic soldiers’ functioning. In addition, further topics in the emotional areas should also be explored during the military service of the autistic population.

## 5 Conclusion

This study suggests that the Roim Rachok Training Course is a holistic vocational intervention, equipping autistic young adults for successful integration into specific military units and the workforce. Post-course, participants reported enhanced adaptive behavior, communication skills, and emotional connections. Consistent improvements across diverse vocational tracks may indicate the training’s positive impact irrespective of individual skill profiles.

## Data Availability

The data that support the findings of this study are available on request from the corresponding author. The data are not publicly available due to privacy restrictions.
