# Supplementary material for: Toward workforce integration: enhancements in adaptive behaviors and social communication skills among autistic young adults following vocational training course
Source: Front Psychol. 2024 Dec 11;15:1392672. doi: 10.3389/fpsyg.2024.1392672 (PMC11668975; doi:10.3389/fpsyg.2024.1392672)
Supplement: Supplementary file 1 [file Table_1.DOCX]

|  | | Appendix A: Vocational interventions focusing on social communication and adaptive behavior enhancement. | | | | | | |
| --- | --- | --- | --- | --- | --- | --- | --- | --- |
| Study | **Program** | | **Type** | **Participants** | **Design** | **Duration/**  **Intensity** | **Measures** | **Results** |
| Baker-Ericzen et al., 2018 | SUCCESS | | Vocational soft skills group | N=8  Age 18-25 y. | Pre-Post | 6-7 months  2/months | *SRS-II*  SSPA  *Brief-A,*  *D-KEFS*  FDLQ | SRS-II self report- T2<T1;  SSPA, BRIEF-A, D-KEFS T2>T1 |
| Gorenstein et al., 2020 | JOBSS | | Vocational soft skills group | JOBSS=22  WLC=11  Age: 18-45 y. | RCT | 15 weeks  1/week | SRS-II  RMET  Employment status | SRS-II self report T2=T1  5 participants were employed |
| Hillier et al., 2007 | Vocational support program | | Supported vocational Internship | N=9  Age 18-36 y. | Follow-Up study | 24 months follow-up  1-8 months support | Employment rate, level of income  self and supervisors  *Assessment Worksheet* | Job skills -T2>T1  7/9 participants remained in their jobs for 2 years. |
| Moody et al., 2022 | PEERS-A Carier | | Vocational soft skills group | N=10  Age:19-30 y. | Pre-post | 10 weeks  2X1.5 h./week | TESS  Employment and intervention surveys | TESS-T2>T1  Improvements in feelings of preparedness for employment |
| Oswald et al., 2018 | ACCESS | | Vocational soft skills group | ACCESS group - 22  WLC group – 22  Age: 18-38 y. | RCT | 19 week  1.5h/week | ABAS-3  SDSS  CSES  ASEBA | ABAS, Self-Determination Performance T2-T1-Research group>WLC group  CSES, ASEBA- T1=T2 |
| Sung et al., 2019 | ASSET | | Vocational soft skills group | N=17  Age: 18-29 | Pre-Post | 8 weeks  1.5h/week | SRS-II  PSSE  PESE | SRS-II (social communication interaction subdomain)- T2<T1  PSSE, PESE- T2>T1 |
| Turner-Brown et al., 2008 | SCIT-A | | Vocational soft skills group | SCIT-A-6  TAU-5  Age 18-55 | quasi-experimental | 18 weeks  50 min/week | FEIT  The Hinting Task  SCSQ  SSPA | FEIT, The Hinting task- T2-T1: SCIT>TAU |
| Wehman et al., 2020 | SEARCH+AS  Vocational support program | | Supported vocational Internship | SEARCH+AS-79  Control- 25  Age: 19-21 | prospective randomized  clinical trial | 9 months | Employment rate  Level of income | Employment rate, Income- SEARCH>Control |

ACCESS- Acquiring Career, Coping, Executive Control, Social Skills.

PESE- Perceived Empathy Self-Efficacy Scale ( Caprara and Steca, 2005).

WLC- waitlist control.
TAU- treatment as usual.
SCIT-A- Social Cognition and Interaction Training.
PSSE- Perceived Social Self- Efficacy Scale (Caprara and Steca., 2005).
D-KEF- The Delis–Kaplan Executive Functioning System (Delis et al., 2001).
FEIT- Face Emotion Identification Test (Kerr and Neale., 1993).
SCSQ- Social functioning- Social Communication Skills Questionnaire (McGann et al., 1997).
The Hinting Task (Corcoran et al., 1995).
SSPA - Social skills performance assessment (Patterson et al., 2001).
CSES -Coping Self-Efficacy Scale (Chesney et al., 2006).
SDSS- Self-Determination Skills Survey (Carter et al., 2013b).
ABAS-3- Adaptive Behavior Assessment System – Adult Form, Third Edition (Harrison and Oakland 2015).
BRIEF-A- The Behavior Rating Inventory of Executive Function–Adult *(*Roth *et al., 2005).*
SSPA -The Social Skills Performance Assessment (Patterson et al., 2001; Baker-Ericzen, 2015a).
SRS-2 Social Responsiveness Scale *(*Constantino & and Gruber, 2012).
JOBSS -Job-Based Social Skills.
RCT – Randomized Controlled Trail
RMET- Reading the Mind in the Eyes Test (Baron-Cohen et al. 1997, 2001).
FDLQ- Functional Daily Living Questionnaire- *(*Baker-Ericzen et al., 2015).
TESS- Test of Employment Social Skills.
PESE- The Perceived Empathy Self-Efficacy Scale (Caprara and Steca, 2005).
ASEBA - Adult Self-Report (ASR) (Achenbach and Rescorla 2003)

Achenbach, TM., Rescorla, L. (2003). Manual for the ASEBA Adult Forms & Profiles. Burlington VT: University of Vermont Research Center for Children,

Youth and Families.

Baker-Ericzen M, Fitch M, Jenkins M, et al. (2015a). *SSPA 2.0 for Autism Spectrum and Related Social Conditions: Employment Version*. Unpublished

Measure, Rady Children’s Hospital, San Diego, CA.

Baker-Ericzen M, Jenkins M, Fitch M, et al. (2015). *Functional Daily Living Skills Questionnaire*. Unpublished Measure, Rady Children’s Hospital, San Diego,

CA.

Baron-Cohen, S., Jolliffe, T., Mortimore, C., & Robertson, M. (1997). Another advanced test of theory of mind: Evidence from very high-functioning adults with

autism or Asperger Syndrome. *Journal of Child Psychology and Psychiatry*, *38*, 813–822.

Baron-Cohen, S., Wheelwright, S., Hill, J., Raste, Y., & Plumb, I. (2001). The “Reading the Mind in the Eyes” Test revised version: A study with normal adults

and adults with Asperger syndrome or high-functioning autism. *The Journal of Child Psychology and Psychiatry and Allied Disciplines*, *42*(2),
241–251.

Caprara GV and Steca P.(2005). Self-efficacy beliefs as determinants of prosocial behavior conducive to life satisfaction across ages. *Journal of Social and*

*Clinical Psychology*. 24(2): 191–217.

Carter, E. W., Lane, K. L., Cooney, M., Weir, K., Moss, C. K., & Machalicek, W. (2013). Self-determination among transition-age youth with autism or

intellectual disability: Parent perspectives. *Research and Practice for Persons with Severe Disabilities*, *38*(3), 129-138.‏

Chesney, M. A., Neilands, T. B., Chambers, D. B., Taylor, J. M., & Folkman, S. (2006). A validity and reliability study of the coping self‐efficacy scale. *British*

*journal of health psychology*, *11*(3), 421-437.‏

Constantino JN and Gruber CP.(2012). *Social Responsiveness Scale, Second Edition (SRS-2)*. Los Angeles, CA: Western Psychological Services

Delis DC, Kaplan E and Kramer JH. (2001). *Delis-Kaplan Executive Function System (D-KEFS)*. San Antonio, TX: Psychological Corporation.

Harrison, P., Oakland, T.(2015). *Adaptive Behavior Assessment System.3*. Torrance, CA: Western Psychological Services.

Kerr SL, Neale JM. Emotion perception in schizophrenia: Specific deficit or further evidence of generalized poor performance? Journal of Abnormal

Psychology 1993;102(2):312–318.

McGann W, Werven G, Douglas MM.(1997). Social competence and head injury: A practical approach. Brain Injury ;11(9):621–628.

Patterson, T. L., Moscona, S., McKibbin, C. L., Davidson, K., & Jeste, D. V. (2001). Social skills performance assessment among older patients with

schizophrenia. *Schizophrenia research*, *48*(2-3), 351-360.‏

Roth RM, Isquith PK and Gioia GA. (2005). *BRIEF-A: Behavior Rating Inventory of Executive Function—Adult Version: Professional Manual*. Psychological

Assessment Resources.
